# New Development of Biomarkers for Gastrointestinal Cancers: From Neoplastic Cells to Tumor Microenvironment

**DOI:** 10.3390/biomedicines6030087

**Published:** 2018-08-13

**Authors:** Jiajia Zhang, Shafat Quadri, Christopher L. Wolfgang, Lei Zheng

**Affiliations:** 1 Departments of Oncology and Surgery, the Sidney Kimmel Comprehensive Cancer Center, the Bloomberg-Kimmel Institute for Cancer Immunotherapy, the Pancreatic Cancer Precision Medicine Center of Excellence Program, the Johns Hopkins University School of Medicine, Baltimore, MD 21287, USA; zhangjiajia@jhmi.edu (J.Z.); cwolfga2@jhmi.edu (C.L.W.); 2 Merck Research Laboratory, Merck & Co., Kenilworth, NJ 07033, USA; shafat.quadri@merck.com

**Keywords:** biomarker, gastrointestinal malignancies, immunotherapy

## Abstract

Biomarkers refer to a plethora of biological characteristics that can be quantified to facilitate cancer diagnosis, forecast the prognosis of disease, and predict a response to treatment. The identification of objective biomarkers is among the most crucial steps in the realization of individualized cancer care. Several tumor biomarkers for gastrointestinal malignancies have been applied in the clinical setting to help differentiate between cancer and other conditions, facilitate patient selection for targeted therapies, and to monitor treatment response and recurrence. With the coming of the immunotherapy age, the need for a new development of biomarkers that are indicative of the immune response to tumors are unprecedentedly urgent. Biomarkers from the tumor microenvironment, tumor genome, and signatures from liquid biopsies have been explored, but the majority have shown a limited prognostic or predictive value as single biomarkers. Nevertheless, use of multiplex biomarkers has the potential to provide a significantly increased diagnostic accuracy compared to traditional single biomarker. A comprehensive analysis of immune-biomarkers is needed to reveal the dynamic and multifaceted anti-tumor immunity and thus imply for the rational design of assays and combinational strategies.

## 1. Introduction

Biomarkers are defined as objective, quantifiable biological indicators of a normal or abnormal process, a condition or disease, or a response to treatment [1]. Prognostic biomarkers allow a selection of patients with a high risk for disease recurrence or rapid progression and help regarding decision making for a treatment regimen. Predictive biomarkers represent an array of indicators that project the patient’s response to the treatment. Currently, biomarkers are genetic, epigenetic, proteinic, or cellular alterations that are inherent to cancer cells, and have been an integral part of individualized cancer care.Glycoproteins, such as carcinoembryonic antigen (CEA) and cancer antigen 19-9 (CA19-9), are classical proteinic biomarkers for disease monitoring [2,3,4]. Biomarkers at the genome level, which are often driver mutations such as *KRAS* and epidermal growth factor receptor (EGFR), have been widely used as a guide for a selection of patients that might benefit from targeted therapies [4,5,6,7]. More recent literature has highlighted *BRCA*, a tumor suppressor gene involved in the repair of double-stranded DNA breaks, as a viable predictive biomarker for response to platinum agents and poly(ADP-ribose) polymerase (PARP) inhibitors [8]. Immunotherapy has revolutionized human cancer treatment by unleashing the potential of the antitumor immune response; however, only 15–20% of patients respond to immunotherapy [9,10]. This underpins the importance of identifying novel biomarkers that can select the patients for immunotherapy. Programmed death ligand 1 (PD-L1) positivity, T cell-inflamed phenotype, and high tumor mutational burden have been reported to enrich the patient populations that benefit from the treatment of immune checkpoint inhibitors (ICIs) [11,12,13,14,15]; however, these makers alone are insufficiently accurate for patient selection across different cancer types. Microsatellite instability (MSI), a pan-cancer biomarker, predicts the response of solid tumors to anti-PD-1/PD-L1 (Programmed death-1/Programmed death ligand 1) blockade, is only found in 1–2% of most of malignancies [12,16]. Preliminary clinical findings showed that the signatures that reflect the composition and metabolites of the gut microbiota would impact the antitumor immune response in patients receiving ICIs, including anti-cytotoxic T-lymphocyte antigen 4 [CTLA-4] and anti-PD-1/PD-L1 antibodies, and have the potential to predict durable clinical responses in non-small cell lung cancer (NSCLC), renal cell carcinoma (RCC), and melanoma; however, the role of the microbiome in predicting the benefits from the ICIs remains unclear for gastrointestinal (GI) cancer [17,18,19,20,21]. A few studies have also suggested the association of members of the gut microbiome with ICI toxicities, but evidence is lacking in the GI setting [21,22]. In addition, biomarkers that can guide the choice of combination immunotherapy are scarce [23]. Quantitative multiplexed approaches, which exert unique advantages in revealing the tumor-immune complexity, may represent a new avenue for biomarker discovery in the tumor microenvironment, especially for combinational therapies targeting the suppressive myeloid/stromal compartment [24,25]. This review summarizes the advances of biomarkers in gastrointestinal malignancies, with a focus on the development of new biomarkers that are of predictive and/or prognostic values in cancer therapies.

## 2. Current Clinical Application of Biomarkers in Gastrointestinal Malignancies

### 2.1. Tumor Markers

The tumor markers currently being used in the clinic are all surrogate markers of malignant diseases (Table 1). CEA is one of the most commonly used tumor markers for gastrointestinal malignancies and a member of the immunoglobulin superfamily [26]. It acts as a mediator for cell adhesion on cancer cells. The overexpression of CEA occurs in >90% of colorectal cancers (CRC) and 60% of other types of cancer, including gastric, lung, and pancreatic cancers [27]; thus, it has been widely used as a serum tumor marker. Its sensitivity and specificity are not high, particularly for the early stages of the disease [28]; therefore, it cannot be used as a biomarker for screening gastrointestinal cancers. However, in the patients with an established disease, the absolute level of the serum CEA correlates with the disease burden and has a prognostic value [29]. In CRC, CEA is the only laboratory test routinely recommended for surveillance. High levels of CEA after surgical resection imply the presence of a persistent disease and the need for further evaluation [30]. The serial measurement of the CEA levels after surgery in patients with colorectal cancer can detect recurrences earlier; nevertheless, this information does not lead to an improved treatment outcome [28]. Currently, the American Society of Clinical Oncology (ASCO) guidelines recommend that the serum CEA levels be obtained in most patients with CRC, so as to aid surgical treatment planning, posttreatment follow-up, and the assessment of prognosis [31]. Another common glycoprotein biomarker is CA19-9, which is used primarily to assess the disease response to therapy or to detect cancer recurrence in patients with gastric cancer, pancreatic cancer, gallbladder cancer, cholangiocarcinoma, or adenocarcinoma of the ampulla of Vater. It is the most useful tumor marker for pancreatic cancer, with sensitivity and specificity rates of 70–92% and 68–92%, respectively [32,33,34]. In addition, an elevated preoperative CA19-9 level is strongly associated with the presence of subradiographic unresectable diseases, and can be used for a selection of patients for staging the laparoscopy [35]. The lack of tumor specificity is the limitation of the currently used tumor markers. Tumor specific markers, particularly those reflecting the tumor biology, are highly demanded.

### 2.2. Targets of Matched Therapies

HER2, a tyrosine kinase receptor belonging to the epidermal growth factor receptor (EGFR) family, is an established prognostic factor and a therapeutic target for gastroesophageal adenocarcinoma [36,37,38,39]. Through the activation of downstream signaling pathways, including RAS/RAF/mitogen-activated protein kinase (RAS/RAF/MAPK) and phosphatidylinositol-3 kinase/protein kinase-B/mammalian target of rapamycin (PI3K/AKT/mTOR), aberrant HER2 amplification or overexpression can lead to uncontrolled cell-cycle progression, cell division, motility, survival, invasion, and adhesion [7]. Approximately 7–38% of gastroesophageal adenocarcinomas have an amplification and/or overexpression of HER2, with a slightly higher positivity in gastroesophageal junction (GEJ), intestinal-type, and well-moderately differentiated tumors [6,7,40,41]. The inhibition of HER2 with the monoclonal antibody trastuzumab in patients with HER2-amplified/overexpressed advanced-stage gastric or esophagogastric-junction adenocarcinomas, confers an improved response rate, progression-free survival, and overall survival when trastuzumab is combined with cisplatin and fluoropyrimidine [5]. Based on the above evidence from the phase III ToGA trial, the current guideline suggests that patients with advanced gastric cancer who are potential candidates for trastuzumab should be screened to determine their HER2 status.

Another biomarker for therapeutic target is c-MET, of which the aberrant expression has been reported in 18–100% of gastrointestinal tumors [42,43]. Activated c-MET signaling results in enhanced cancer cell proliferation, survival, and invasion, and is an independent prognostic factor for inferior survival [44,45,46]. Several monoclonal antibodies and small-molecule inhibitors of c-MET have been evaluated in clinical trials, however, most of the phase III trials of MET inhibitors showed negative results for gastric cancer. In hepatocellular carcinoma, although encouraging results were reported in phase II studies [47,48,49], the phase III trial failed to show an improvement in the overall survival compared with the placebo in patients with c-MET-positive advanced hepatocellular cancer (HCC), casting doubt on the role of MET inhibition as a viable therapeutic strategy [50,51]. Therefore, the development of biomarkers for therapeutic target is still a challenge.

In metastatic CRC, the *KRAS* mutation status has been widely reported as a prognostic and predictive biomarker [52]. *KRAS* mutations can be identified in 12–75% of colon cancers and are independently associated with a worse prognosis in the majority of the studies, albeit not all of the studies [53,54]. As the *RAS* oncogene is located at the downstream of the EGFR signaling pathway, the *RAS* mutations can lead to an activation of the pathway, even if the EGFR is blocked [55]. Thus, the *KRAS* mutation status is a biomarker for unresponsiveness to anti-EGFR therapy. Interestingly, there is a bias toward the right-sided CRC for the *KRAS* mutation, this may partially explain an inferior survival and poor response to targeted therapy with EGFR inhibitors for the right proximal CRC compared to the left colon CRC [56]. The characteristics of the HER2, c-MET, and KRAS expression in GI cancers are summarized in Table 2.

### 2.3. Mismatch Repair Genes

Mismatch repair (MMR) gene products function to repair the nucleotide base mispairings and small insertions or deletions that occur during DNA replication [57,58]. Thus, the MMR-deficient tumors could accumulate hundreds to thousands of somatic mutations, regardless of their cell of origin. It has been implicated in the pathogenesis of the hereditary nonpolyposis colorectal cancer syndromes, as well as a variety of different sporadic cancers. MMR-deficiency is present in 15–20% of all colorectal cancers (CRCs), 8.5–20% of gastric cancers, 3–7% in esophageal/GEJ adenocarcinomas, and 2–3% of pancreatic cancers [12,16,59,60]. MMR-deficiency has been shown to be positively prognostic for survival in patients with colon, gastric, and pancreatic cancers [57,60,61]. It could also serve as a potential predictive marker for a lack of efficacy of fluoropyrimidine based adjuvant chemotherapy in gastric cancer and colon cancer [62,63,64]. Importantly, MMR deficiency is a pan-cancer predictor for response to anti-PD-1/PD-L1 blockade therapies [65]. It is hypothesized that tumors with an MMR deficiency are enriched with missense mutations that are presented as neoepitopes to T cells, which are subsequently targets of anti-PD-1/PD-L1 therapies. Le et al. reported a phase II clinical trial of progressive metastatic carcinoma with or without MMR deficiency, and revealed significantly increased somatic mutations per tumor in the MMR–deficient tumors compared with the MMR-proficient tumors (mean, 1782 vs. 73). The MMR deficient patients had a remarkably increased immune-related objective response rate (40% vs. 0%) and prolonged immune-related progression-free survival rate (78% vs. 11%), compared to their counterparts [16]. In an expanded cohort of 86 patients with MMR-deficient tumors, the objective radiographic responses were noted in 53% of the patients (46 of 86 patients; 95% CI, 42–64%), with 21% (*n* = 18) achieving a complete radiographic response. Disease control (measured as partial response + complete response + stable disease) was achieved in 66 (77%) of the 86 patients (95% CI, 66–85%) [12]. This led to the approval from the Food and Drug Administration (FDA) for testing MMR-deficiency in order to identify the candidate patients who may benefit from a second-line PD-1 pathway blockade, regardless of the tumor types [66]. Of note, this is the first and only FDA approved pan-cancer biomarker for immune checkpoints blockade. Clinical trials investigating its role as predictive biomarkers in the first-line and (neo)adjuvant settings are ongoing [67,68].

## 3. New Development of Biomarkers

### 3.1. Biomarkers in Tumor Microenvironment

#### 3.1.1. PD-L1 Expression

As above described, PD-1, which is expressed on activated lymphocytes, including T cells, B cells, and natural killer cells, limits the T cell effector functions within tissues. By upregulating the ligands for PD-1 (PD-L1), tumor cells induce the apoptosis of the effector T cells [69,70]. The reported incidence of PD-L1 expression ranges differently in the different tumor types (14–100%), whether or not these tumors respond to anti PD-1/PD-L1 treatment [71,72,73,74,75,76]. Early studies have suggested that PD-L1 positivity enriches the patient populations that can benefit from PD-1/PD-L1 axis inhibition [77,78], with the hypothesis that pre-existing immunity suppressed by PD-1/PD-L1 could be re-invigorated on antibody treatment with checkpoint blockade. However, more studies questioned the accuracy of PD-L1 as an effective predictive biomarker. In the recent phase III trials testing the adjuvant anti-PD-1 in resected stage III melanoma, the first-line anti-PD-1 antibody in combination with chemotherapy in metastatic NSCLC, and the combination of anti-PD-1 and anti-CTLA4 antibodies in NSCLC with a high mutational burden, the benefit of immunotherapy did not correlate with the PD-L1 expression level [79,80,81]. In the Keynote 059 trial, objective responses and complete responses (CRs) were observed in both the PD-L1-positive and negative gastric and gastroesophageal adenocarcinoma patients who had previously received at least two lines of treatment [82]. The PD-L1-positivity was defined as a combined positive score (CPS) ≥1%, where CPS is the number of PD-L1 staining tumor cells, lymphocytes, and macrophages divided by the total number of viable tumor cells multiplied by 100, using PD-L1 Immunohistochemistry (IHC) 22C3 pharmDx immunohistochemistry. Although the objective response rate (ORR) seemed higher in the patients with PD-L1–positive compared with the PD-L1–negative tumors (23 of 148 [15.5%] vs. 7 of 109 [6.4%], respectively), the patients with PD-L1–negative tumors also experienced objective responses, including CR in three patients (2.8%) [82]. Nevertheless, this study has gained the FDA approval of using PD-L1 positivity (Table 1) at the 1% cutoff as a biomarker to select patients with metastatic gastric and gastroesophageal adenocarcinoma for the treatment of pembrolizumab [83]. In the Asian population, Nivolulab was approved for the treatment of the unresectable, advanced, or recurrent gastric cancer that has progressed after using conventional chemotherapy, based on the results from the phase III ATTRACTION-2 trial, regardless of PD-L1 status. In hepatocellular cancer (HCC), the report from the phase I/II trial suggests that ICIs elicited a promising response rate of 16–19% (49/255) in advanced HCC, but the response rate to the ICIs did not differ according to the PD-L1 expression status [82,83,84,85]. In PDACs, reports of the PD-L1 expression vary from 12–90%, however, single agent anti-PD1 treatment has shown no efficacy, except for MMR deficient patients, regardless of PD-L1 status [86].

Therefore, evolving evidence suggests that PD-L1 testing alone is insufficiently accurate to predict patient response to immunotherapy, although it may be used in some GI cancers to enrich the patients that may more likely benefit from anti-PD-1/PD-L1 antibodies (Table 3). Several factors may explain the heterogeneity of the predictive values for the PD-L1 expression, including differences in the PD-L1 IHC assay platforms and detection antibodies, differing IHC cutoffs, tissue preparation, processing variability, primary versus metastatic lesions, oncogenic versus induced PD-L1 expression, and the staining of tumor versus immune cells [42,47,75,87,88]. It should be noted that the PD-L1 expression measured in the clinical assays may only represent a snapshot of the dynamic and multifaceted immune cells and their complex interaction with neoplastic cells. A comprehensive characterization of the tumor microenvironment is necessary to adequately assess the strength of PD-L1 in predicting the immune response to anti-PD-L1/PD-1 therapies.

#### 3.1.2. Tumor Infiltrating Lymphocyte

Tumor infiltrating lymphocytes (TILs) represent a potent machinery of the adaptive immunity that has the antitumor potential. TILs have been shown to be associated with improved prognoses and response to immunotherapy in various cancer type (Table 3) [24,44,45,46,89,90,91]. In colorectal cancers, the type, density, and location of the immune cells, specifically the cytotoxic and memory T cells, has been reported to be a better predictor of survival than (Union for International Cancer Control ) UICC-TNM staging 89]. Among the tumors with similar degrees of T cell infiltration, those with the greatest proportion of CD103+ memory T cells have the best prognosis [92]. To standardize the method of evaluating TILs in CRC, a new method that measures the area occupied by mononuclear cells over the stromal area on hematoxylin and eosin (H-E)-stained sections was proposed. The results from such a method confirmed the density of TILs as a useful prognostic factor in CRC [93]. In Epstein–Barr virus (EBV)-associated gastric cancer an association between a high percentage of TILs, low intratumoral PD-L1 expression, and longer disease-free survival (DFS) was demonstrated [94]. A meta-analysis on 23 relevant studies of 3173 hepatocellular carcinoma (HCC) patients showed that high levels of CD8+ and CD3+ TILs had a better prognostic value on the overall survival (OS), yet high levels of FoxP3+ TILs had a worse prognostic value on OS and DFS/Relapse-free survival (RFS), implicating that TILs may serve as a prognostic biomarker in HCC [95]. A TIL density of ≥5% was reported to be associated with a better objective response as well as the progression free survival (PFS) in NSCLC patients treated with Nivolumab [96]. Recently, a T cell inflamed expression score utilizing 18 gene signatures was shown to be significantly associated with a Pembrolizumab response in gastric/GEJ cancer [97,98]. A significant but nonlinear association was found between the T cell-inflamed gene expression score and PD-L1 expression. These results suggest the potential for a T cell-inflamed gene expression profiling score in association with the PD-L1 expression as biomarkers for treatment selection in gastric/GEJ cancers. In pancreatic cancer, variable frequencies of endogenous CD8+ T cells, CD4+Foxp3− T cells, and CD4+Foxp3+ regulatory T cells (Treg) were reported. Notably, these T cells were enriched within CD20+ lymphoid aggregates, with a trend toward longer survival in those patients with tumoral Tertiary lymphoid structures [99]. In our cohort of 24 pancreatic ductal adenocarcinomas receiving neoadjuvant GVAX^®^vaccination, which is a tumor vaccine composed of autologous tumor cells genetically modified to secrete granulocyte–macrophage colony-stimulating factor (GM-CSF), the ratios of CD8+ T cell to CD68+ T cell are favorable predictors of survival, as reported in other malignancies [100,101]. Nevertheless, the above results need to be confirmed in future trials and more studies on whether and how TILs or effector T cells can be used to predict the response to immunotherapy in gastrointestinal malignancies are warranted.

#### 3.1.3. Immunosuppressive Myeloid Cells

Tumor-associated myeloid cells not only create a suppressive or anergic environment by blocking T cell functions and proliferation, but also accelerate tumor growth by promoting cancer stemness, angiogenesis, stroma deposition, epithelial-to-mesenchymal transition, and metastasis [102]. The accumulation of the intratumoral and circulating myeloid derived suppressive cells (MDSCs) has been shown to be associated with disease progressiveness and prognosis in pancreatic adenocarcinoma, hepatocellular carcinoma, and gastric cancer (Table 3) [102,103,104,105,106]. In our study evaluating 24 pancreatic ductal adenocarcinomas from patients who received neoadjuvant GVAX vaccination, although, essentially all of the tumors have induction of TILs and PD-L1 expression, the survival of patients is correlated with the infiltration of myeloid cells [76]. Nevertheless, myeloid cells are also critical for an innate immune response. It is unlikely that a single myeloid marker would be able to predict the immune response. Multiplex biomarker assays will need to be developed for characterizing immunosuppressive myeloid cells before a clinical assay can be used for predicting their response to immunotherapy.

#### 3.1.4. Intratumoral Stroma

**Figure 1:**
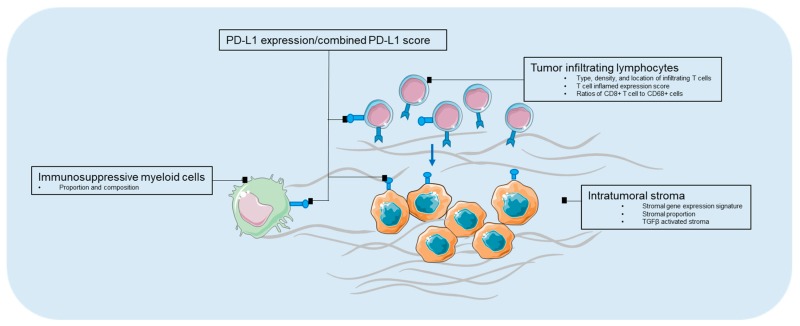
Biomarkers in tumor microenvironment. PD-L1—programmed death-1 ligand-1.

The intratumoral stromal (ITS) proportion, composition, and activation status represent another array of biomarkers for the disease prognosis. Stromal proportion, quantified by histopathological microscopy analysis of the conventional hematoxylin and eosinstained slides, has been reported to be independently associated with poor prognosis in several types of cancers, including gastric cancer, esophageal, and colon cancers (Table 3) [107,108,109,110,111]. Wu et al. showed that the stromal gene expression signature as well as the ITS proportion quantified by morphometry in tissue sections of patient samples, correlated with the survival of gastric cancer patients in multiple independent cohorts. Measuring the relative amount of ITS may enable the identification of subgroups of gastric cancer patients that benefit from stroma-directed therapies [112]. More recently, transforming growth factor beta (TGFβ) activated stroma was found to represent a primary mechanism of immune evasion that engenders T cell exclusion and primary resistance to anti-PD-1–PD-L1 therapy in microsatellite-stable (MSS) CRC. In murine models, the authors showed that the inhibition of TGFβ signaling in the stoma with a TGF-β receptor 1 (TGF-βR1) specific inhibitor could lead to a potent anti-tumour cytotoxic T cell response and prevent metastasis [113]. Admittedly, there are promising applications in immunotherapies targeting intratumoral stroma and in combination with immune checkpoint inhibitors, however, the identification of accompanying predictive features from intratumoral stroma to enrich for populations that can benefit from combinational therapies are crucial. A summary of biomarkers in the tumor microenvironment is depicted in Figure 1.

### 3.2. Biomarkers in Tumor Genomics

#### 3.2.1. Targeted Gene Panels

Targeted gene sequencing is an emerging approach for identifying potentially targetable genomic biomarkers and matching them for treatments. However, a number of challenges remain. For example, the Memorial Sloan Kettering Cancer Center has developed an (IMPAC) targeted gene sequencing panel, which included 341 genes initially, and has expanded to 410 cancer-associated genes [114]. As reported, 10,945 tumors from 10,366 patients with advanced cancer were sequenced. Eleven percent of the patients were enrolled in a genomically matched clinical trial. Among the 10,366 patients, 338 patients had pancreatic cancer. Five of these pancreatic cancer patients died before the results were finalized. Potentially actionable findings were noted in 26% of these pancreatic cancer patients. Nevertheless, only three of the 225 patients (1%) who would need treatments received matched therapy based on the sequencing results [115]. Two had no benefit and one had an unknown response. Therefore, the practical application of molecular results to guide individual patient treatment is currently limited in patients with pancreatic adenocarcinoma.

#### 3.2.2. Mutational Burden

The tumor mutational burden (TMB) has been shown to be significantly associated with a clinical benefit to immune checkpoint blockade in various cancer types [6,11,13,14,15,35,116]. However, most of the GI cancers have low mutation burdens [5], except those with MMR-deficiency. In a cohort of 1375 patients across various GI tumors, colon cancer was reported to have the highest TMB (mean: 11.6 and 9.9 mutations [mut]/megabase [MB]), whereas biliary cancers and pancreatic adenocarcinomas had the lowest TMB (mean: 5.7 and 4.9 mut/MB) [117]. Using a cut-off of 17 mut/MB to define high vs. low TMB, the high TMB was seen most frequently in right sided colon cancer (12%), gastric cancer (11%), and anal cancer (8%), and least frequently in pancreatic cancer (1.3%) and esophageal squamous cell carcinoma (0%). In addition, primary tumors, MSI-H and/or MSS with *POLE* mutations were observed to have a higher TMB. Those with a higher frequency of somatic mutations and tumor-specific neoantigens were found to have more abundant infiltration of CD8+ T lymphocytes and a higher expression of regulatory molecules (CTLA-4, PD-1, Lymphocyte-activation gene 3[LAG-3] and indolamine 2,3-dioxygenase 1 [IDO1]) [118].

### 3.3. Biomarkers in Liquid Biopsies

A tumor tissue biopsy would be necessary to establish the diagnosis; however, it would not be feasible for monitoring the treatment response [119]. The analysis of biomarkers from peripheral blood, including the circulating tumor DNA (ctDNA), circulating tumor cells (CTC), and exosomes, using a noninvasive approach known as liquid biopsy, has emerged as a way to overcome the restrictions of tumor tissue biopsies and has exhibited a great potential of being used to detect the recurrence and measure the treatment response [120].

#### 3.3.1. ctDNA

ctDNAs are predominantly released as a result of the apoptosis or necrosis of actively growing cancer cell, but can also be secreted directly from the circulating tumor cells [121]. Notably, ctDNA can maintain tumor-specific genomic aberrations, including point mutations in tumor suppressors and oncogenes, copy number variants, DNA methylation patterns, and chromosomal rearrangements, providing a comprehensive genomic profiling for tumor evolution and dynamics disease monitoring [119]. In CRC, ctDNA has been shown to successfully gather the real-time molecular evolution in patients treated with EGFR targeted therapy [122,123]. The ctDNA analyses can not only identify genetic alterations that are likely to be responsible for resistance to EGFR blockade, but also can guide the selection of rare populations of patients who are likely to respond to targeted agents [124]. Changes of circulating tumor DNA (ctDNA) levels during therapy might also be an indicator of clinical efficacy with ICIs. In a small prospective pilot study (*n* = 15), the ctDNA levels at week eight showed synchronous changes with tumor size and as well as an association with PFS in various cancer types [125]. In the chemotherapy setting, the RAS/BRAF mutations detected in ctDNA correlated with a worse PFS in the metastatic CRC patients (*n* = 27) treated with first-line chemotherapy [126]. However, the role of ctDNA in predicting the response to treatment needs to be validated in a larger population.

#### 3.3.2. CTC

CTCs are surrogates of tumor cells in the bloodstream. CTC prevalence differs with cancer type and stage. In patients with metastatic GI malignancies, CTC could be detected in 30–66% of patients [127], and its presence has been shown to correlate with decreased OS or decreased PFS [128]. The value of CTCs as a therapeutic target to monitor the treatment response and detect relapse has also been reported [129]. Nevertheless, the prognostic and predictive role of CTC is not established in a non-metastatic setting, given the scarcity of CTC in this patient population [130,131]. On the other hand, CTC recently demonstrated its value in monitoring the response to immunotherapy. In a prospective cohort of 49 metastatic melanoma patients treated with ICI, a decrease in an RNA signature score of CTC within seven weeks of therapy correlated with a marked improvement in PFS (hazard ratio [HR], 0.17; *p* = 0.008) and overall survival (OS) (HR, 0.12; *p* = 0.04) [132]. The promising results support the rationale to apply CTCs in monitoring the tumor burden in other cancer types such as GI cancers.

Thus far, despite the interesting and promising results from small cohorts of studies on liquid biopsy approaches as predictive or prognostic biomarkers, there is insufficient evidence of clinical utility of the majority of ctDNA/CTC assays in either advanced cancer or early-stage cancer [133]. Discordance exists between the results of different platforms [134]. Prospective trials in large populations will be required to establish the clinical utility of ctDNA and CTC.

#### 3.3.3. Exosomes

Exosomes are endosome derived extracellular vesicles (EVs) ranging in 30–120 nm [135]. They carry a cargo of proteins, metabolites, RNAs (mRNA, miRNA, long non coding RNA), DNAs (mtDNA, ssDNA, and dsDNA), and lipids [136], and represent an important source as a biomarker from liquid biopsies. In pancreatic cancer, the glypican-1 (GPC1)+ endosomes were reported as a diagnostic biomarker to distinguish healthy subjects and patients with a benign pancreatic disease from patients with early- and late-stage pancreatic cancer, with absolute specificity and sensitivity. The levels of the GPC1(+) endosomes correlated with the tumor burden and the survival of pre- and post-surgical patients [137]. A rapid, highly sensitive, and widely usable detection method based on the amplified luminescent proximity homogeneous assay, using photosensitizer-beads for cancer cell-derived EVs was proposed, with the utilization of monitoring the circulating EVs with the antigen CD147 for the detection of colorectal cancer [138]. Although endosomes held great promise for non-invasive early detection and target for potential therapeutics, it has several limitations. One of the biggest challenges in exosome biology is how to accurately measure the quantity and purity of the exosomes. Only a small subset of EVs carry the relevant communication content, thus its actual efficiency may be difficult to detect. In addition, more knowledge of the specific markers of the EV subtypes and fundamental roles of each type of EV is required to better inform their utilization in various disease settings. The advantages and limitations of various liquid biopsy approaches are summarized in Table 4.

## 4. Prospective

There are only a handful of biomarkers used in clinics for the management of GI malignancies. Although many new biomarkers have been identified for GI malignancies, their clinical assays have not been validated. On the other hand, biomarker assays are highly demanded by a selection of patients for appropriate treatment. Nevertheless, such a biomarker is often not conceived until a clinical trial of experimental therapeutics fails to meet its endpoint because of a lack of patient selection. The development of experimental therapeutics will not be advanced until an adequate biomarker assay is established. Therefore, in the future, biomarker development should be done in parallel with drug development. Whenever a potential therapeutic target is identified, a companion biomarker assay should be developed. For immunotherapy, a single biomarker is often not sufficient in predicting the treatment response. A comprehensive analysis of immune-biomarkers can not only provide the rational design of combination immunotherapy, but can also identify multiple immune-biomarkers, and subsequently develop a multiplex assay to co-evaluate multiple immune-biomarkers. In addition, recent research on ion channels and aquaporins have suggested their function as possible modulators of important processes in gastrointestinal carcinogenesis, including colorectal, pancreatic, gastric, and esophageal cancers, as well as their potential as new cancer biomarkers once appropriately validated [141,142,143].

## Figures and Tables

**Table 1:** Major molecular markers in clinical application.

Molecule	Tumor Type	Implication
**Tumor markers**		
CEA	Colorectal, gastric, and pancreatic cancers	Indicating residual disease, progressive, or recurrent disease
		Measuring treatment response
CA19-9	Pancreatic cancer	Indicating residual disease, progressive, or recurrent disease
		Measuring treatment response
**Targets of matched therapies**		
*HER2*	Gastric or esophagogastric-junction cancers	Selecting for targeted therapy
*KRAS*	Colorectal, gastric, and pancreatic cancers	Predicting for treatment unresponsiveness
**Mismatch repair Genes**		
*MMR*	Solid tumors	Predicting for treatment responsiveness
**Biomarkers in tumor microenvironment**		
PD-L1 expression	Gastric cancer	Enriching patient population responding to anti-PD-1/PD-L1 therapies

CEA—carcinoembryonic antigen; MMR—mismatch repair; PD-L1—programmed death ligand 1; PD-1—programmed death-1.

**Table 2:** Characteristics of HER2, c-Met, and KRAS expression in gastrointestinal (GI) cancers.

Molecule	Genomic Alterations	Pathways Involved	Cancer types	Treatment
HER2	Amplification/ overexpression	Activation of the MAPK and the PI3K/AKT axis	Gastric or esophagogastric-junction cancers	Monoclonal antibodies (e.g., cetuximab and trastuzumab)
c-MET	Amplification/ overexpression	Activation of GRB2-SOS–RAS–MAPK, the PI3K/AKT axis, and STAT3 pathway	Colorectal cancer, gastric cancer, pancreatic cancers and hepatocellular carcinoma	Monoclonal antibodies (e.g., rilotumumab, ficlatuzumab, and TAK-701); Tyrosine kinase inhibitors (e.g., tivantinib, cabozantinib, and crizotinib)
KRAS	Activating mutation within catalytic RAS domain	RAS–RAF–MEK	Colorectal cancer	Downstream pathway inhibitors (e.g., *MEK* inhibitors selumetinib and trametinib)

MAPK—mitogen-activated protein kinase; GRB2—growth factor receptor-bound protein 2; STAT—signal transducer and activator of transcription; PI3K—the p85 subunit of phosphatidylinositol 3-kinase; SOS—son of sevenless homologue 1.

**Table 3:** New development of biomarkers.

Molecule	Tumor Type	Implication
**Biomarkers in tumor microenvironment**		
PD-L1 expression	Other cancer types, except gastric cancer	Enriching patient population responding to anti-PD-1/PD-L1 therapies
Tumor infiltrating lymphocyte	Colon and gastric cancers	Indicating good prognosis
Immunosuppressive myeloid cells	Pancreatic, hepatocellular, and gastric cancers	Indicating poor prognosis
Intratumoral stroma	Gastric, pancreatic, esophageal, and colon cancers	Indicating poor prognosis
**Biomarkers in tumor genomics**		
Targeted gene panels	Pan-cancer	Selecting patients for targeted therapies
Mutational burden	Pan-cancer	Enriching patient population responding to anti-PD-1/PD-L1 therapies
**Biomarkers in liquid biopsies**		
ctDNA/CTC/Exosomes	Pan-cancer	Indicating residual disease, progressive, or recurrent disease
		Measuring treatment response

CTC—circulating tumor cells.

**Table 4:** Advantages, disadvantages of ctDNA, CTC, and exosome as biomarkers.

Approaches	Advantages	Disadvantages	References
ctDNA	Higher sensitivity; quick renew/short half-life; maintain tumor-specific genomic aberrations	Not suitable for functional assay, noises from normal cell-free DNA, challenges in methods’ standardization	[134,138,139]
CTC	Allow morphological/molecular/functional study; potentials for therapeutic targets	Low specificity, particularly in early stage setting; challenges in methods’ standardization limited capture techniques	[121,127,128,134,140]
Exosomes	Higher sensitivity; higher serum concentration; diverse EV contents; Potential for therapeutic targets	Isolation and purification of exosomes; specific exosome marker to identify subset of EVs; not suitable for functional assay; challenges in methods’ standardization	[135,136,137]

EV—extracellular vesicles.
